# Longitudinal Study of Fecal Microbiota in Calves with or without Diarrhea Episodes before Weaning

**DOI:** 10.3390/vetsci9090463

**Published:** 2022-08-29

**Authors:** Pau Obregon-Gutierrez, Jaume Bague-Companys, Alex Bach, Virginia Aragon, Florencia Correa-Fiz

**Affiliations:** 1 Unitat Mixta d’Investigació IRTA-UAB en Sanitat Animal, Centre de Recerca en Sanitat Animal (CReSA), Campus de la Universitat Autònoma de Barcelona (UAB), Bellaterra, 08193 Barcelona, Spain; 2 IRTA, Programa de Sanitat Animal, Centre de Recerca en Sanitat Animal (CReSA), Campus de la Universitat Autònoma de Barcelona (UAB), Bellaterra, 08193 Barcelona, Spain; 3 OIE Collaborating Centre for the Research and Control of Emerging and Re-Emerging Swine Diseases in Europe (IRTA-CReSA), Bellaterra, 08193 Barcelona, Spain; 4 Marlex Recerca i Educació, 08173 Barcelona, Spain; 5 Institució de Recerca i Estudis Avançats (ICREA), 08007 Barcelona, Spain

**Keywords:** fecal microbiota, diversity dynamics, calf, cattle, diarrhea

## Abstract

**Simple Summary:**

Animal production is searching for ways to reduce antimicrobial use, and the best way is to avoid their use by maintaining the health of the animals. The microbiota is involved in the host health, and when the fecal microbiota was analyzed in calves that developed or not diarrhea, differences linked to the health status were detected. While changes in the fecal microbiota were observed with time (during the first 2 months of age) in all the calves, the microbiota from the healthy animals presented an earlier stabilization and some changes in low abundant bacteria, which may play a role in the subsequent health status of the animals. Bacteria classified in the families *Coriobacteriaceae* and *Phyllobacteriaceae*, and the bacterium *Epulopiscium* were found in the core of the microbiota of the healthy calves (calves that did not have diarrhea) possibly with a protective probiotic effect. On the other hand, several bacteria, such as *Lachnospira*, *Neisseria* and *Solibacillus*, were found only in the core of the microbiota obtained from calves that had diarrhea, indicating that they could be linked to a higher predisposition to suffer diarrhea. These results can help in the development of new probiotics to promote gut health in calves.

**Abstract:**

The microbiota plays an important role in the development of diarrhea in pre-weaned calves. The characterization of the fecal microbiota in health and disease can be critical to unravel the bacterial dynamics associated with diarrhea and help with its prevention and control. In this study, we aimed to detect changes in the fecal microbiota of calves that experienced early-life diarrhea episodes. Fecal samples were taken from calves remaining healthy and calves with an episode of diarrhea during the study. We sampled at arrival (12 days of age) and after one and two months of life; also, at the time of the diarrhea episode for the diarrheic calves (day 17). Samples were processed to extract total DNA, submitted to 16S rRNA gene sequencing, and bioinformatically analyzed to infer the bacterial populations. Microbiota changes through time were reported for both groups. However, we detected an earlier stabilization in the healthy group. Moreover, we detected changes within low abundant taxa that may play a role in the subsequent health status of the animals. The fecal microbiota of healthy and diarrheic calves showed different dynamics in the diversity through time that may be the reflections of the variations within low-abundant taxa.

## 1. Introduction

Diarrhea in pre-weaned calves is among the biggest concerns in the cattle industry, being responsible for great economic losses [1], with consequences that go beyond poor performance or mortality, which was quantified to be around 10% [2]. Despite being a multi-etiological disease [1,3], calf diarrhea is generally treated with antimicrobials [2], whose usage is sometimes imprudent in animal production [4]. Antimicrobials produce negative aspects, such as the appearance of bacterial resistances [4] or the disruption of health-associated microbial communities, which can, in turn, lead to more severe diseases [5,6]. Although antimicrobials have proven to be essential, their misuse and overuse is still one of the most important concerns in animal production, but also in global health [4,7], making it vital to find alternative ways to control and prevent diseases.

The gut microbiota, that is, the microorganisms that inhabit the gastrointestinal tract of animals, has an important role in animal health. The gut microbiota has been shown to have many positive effects on its host, with many implications for immune system maturation, metabolic and physiological functions and protection against pathogens [8]. This may be particularly important at early life stages when the microbiota is still unstable [8] since the proper development and maintenance of these healthy microbial communities promote the good health status of the animals later in life and therefore it may reduce the need for antimicrobial treatments [6,8]. In the case of calves, several studies reported the benefits of developing a healthy gastrointestinal microbiota in relation to disease development [9,10,11]. The role that microbial communities may play in calf diarrhea has been explored, showing that higher diversity is linked to a healthier status [6,9], and detecting specific taxa as disease markers [6,9,12]. More information that allows the identification of the components associated with healthy microbiota during the preweaning stage together with the perturbations detected during disease is still needed to better characterize the role of the gut microbiota at this critical period. Furthermore, the study of the microbiota-diarrhea interaction in calves early in life is of particular interest [13], as a healthy gastrointestinal tract seems to exert positive long-term effects on their health [6]. Therefore, understanding the gastrointestinal microbiota of calves during the first months of life and its possible connection with early life diarrhea may be a key point in the control of this pathology at this critical moment in calves’ lives, but also to help improve long-term animal health and welfare.

In this study, we compared the bacterial composition of feces from calves that remained healthy during the pre-weaning period with those that experienced diarrhea and were consequently treated with antibiotics, with the aim of identifying variations between the two groups.

## 2. Materials and Methods

### 2.1. Study Design

For a period of two months, 588 healthy female calves that entered a commercial contract heifer operation (Rancho las Nieves, Mallén, Spain), arriving from different farms, were enrolled in a sampling scheme consisting of collecting fecal samples at three time points: at arrival, when calves were on average 12 days-old (12 ± 2.26), considered herein as day 12; at one month (day 33 ± 1.61); and at two months (day 61 ± 1.54) of life. At the farms of origin, calves were fed with colostrum for 1 or 2 days and then a milk replacer (which, on most occasions, was the same as the one used in the contract heifer operation). No antibiotics were used in the farms or origin. Upon arrival, calves were 43.9 ± 7.1 kg in body weight and 84 ± 3.6 cm in height. The body weight was determined using an electronic scale. On a daily basis, all calves were checked by the same veterinarian. Calves were fed with a milk replacer (22 % protein, 18% fat; Nukamel, Belgium) and a starter feed (20% CP, 15% NDF on a DM basis). Those animals that remained healthy were classified as H group, while those that had an episode of diarrhea during the study were classified as D group. Fecal scores were recorded daily based on a 1 to 5 system: 1 = normal, thick in consistency; 2 = normal, but less thick; 3 = abnormally thin but not watery; 4 = watery; 5 = watery with abnormal coloring [14]. Only calves with a fecal score of 4 or 5 for three consecutive days were treated with antibiotics (and were included in the study). The total number of calves in the H group was 455 arriving from 110 different farms, and the total number of calves in D was 133 group arriving from 92 different farms. Calves undergoing any other afflictions, such as pneumonia or omphalitis, were discarded from the study. Calves with diarrhea were treated with two subcutaneous injections of 2.5 mg of cefquinome sulfate per kg of weight spaced 48 h if diarrhea was still present (score of 4 or 5). All samples were named according to the group they belonged followed by the mean age of the animals (H12, H33, H61 and D12, D33 and D61). In addition, the D group was sampled at the moment that calves experienced the diarrhea episode, ranging from 16 to 18 days of life (day 17 ± 0.82). Then, 10 calves from each group were randomly selected and their fecal samples were processed to recover DNA. However, due to technical issues during genomic DNA extraction and sequencing, the final number of samples per group was n = 7 for H and n = 6 for D group. The ages and the farm of origin of the selected calves at different sampling times are depicted in Table 1.

### 2.2. Sampling, DNA Extraction and 16S rRNA Gene Sequencing

Fecal swabs were collected from fecal material obtained via rectal palpation from each calf and immediately transported to the lab and stored at −20 °C, where swabs were resuspended in 500 μL of PBS and stored at −80 °C. Genomic DNA extraction was performed with a Nucleospin Blood kit (Machinery Nagel, GmbH & Co., Düren, Germany) following the manufacturer’s protocol, and the DNA quantity and quality were assessed with BioDrop DUO (BioDrop Ltd., Cambridge, UK).

A 16S rRNA gene library was prepared from the total extracted DNA and sequenced at Servei de Genòmica, Universitat Autònoma de Barcelona, (Illumina pair-end 2X250 bp, MS-102-2003 MiSeq Re-agent Kit v2, 500 cycle). The size of the amplicons was verified on a Bioanalyzer DNA 1000 chip (Agilent), as expected amplicon lengths using Illumina recommended primers were around 460 bp. Finally, the sequences corresponding to variable regions V3-V4 of the 16S rRNA gene were sorted into samples and used as input for bioinformatic analysis. 

### 2.3. Microbiota in Silico Analysis

The fecal microbiota composition was analyzed with quantitative insights into microbial ecology (QIIME) 2 software version 2021.4 [15], through the following pipeline. At first, raw reads were imported in QIIME2 (*q2 import tools*), whose quality was assessed using the *q2 demux* plugin. Secondly, reads were denoised with DADA2 [16], obtaining the Amplicon Sequence Variants (ASVs) to be analyzed. This step was performed separately for each of the runs to deal with possible batch effects and then merged into a single dataset afterward. To remove nonprokaryotic features, an extra quality filtering step was performed with VSEARCH [17] using 65% identity and 50% query cover within the *q2 quality control* plugin [18] to remove ASVs not matching Greengenes database vs. 13.8 [19] clustered with 88% identity (available at https://docs.qiime2.org/2020.8/data-resources/, accessed on 21 January 2022). To avoid the methodological issues appearing when working with variable sampling depths [20], the microbial composition was assessed at a normalized depth of 58,940 for all samples, corresponding to the lowest read-count sample, where the mean number of reads per calf was 301,995. The read counts for each sample can be found in Supplementary Table S1. Sequences were aligned using MAFFT [21], and hypervariable positions were masked [22] with the *q2 alignment* plugin. A phylogenetic tree was built with Fastree [23]. 

In order to study the changes in the fecal microbiota of the two groups of calves in the first two months, we assessed the alpha (within each sample) and beta diversity (between samples) both over time and according to the health status of the animals. Alpha diversity was estimated with Chao [24] and Shannon [25] indexes, used to compute alpha group significance by pairwise non-parametric t-tests (999 random permutations) with *q2 diversity alpha-group-significance* plugin [26]. Beta diversity distance matrices were computed with the *core-metrics* plugin and used to perform principal coordinate (PCoA) analysis [27,28], which were visualized using Emperor [29]. The used metrics for qualitative and quantitative analyses were Jaccard [30] and Bray Curtis [31] dissimilarity indexes, respectively. Adonis test function from the vegan package was used to quantify the group variation in R software [32], which significance was calculated by PERMANOVA pairwise test, with 999 random permutations using *q2 diversity beta-group-significance* plugin [33]. Taxonomical assignment to representative sequences was done with the Python module for machine learning, scikit-learn, using the pre-trained naïve Bayes classifier [34], previously trained against V3-V4 regions from 16S rRNA gene Greengenes (13.8 version) pre-clustered at 99% sequence identity, in order to improve its performance [35]. Differently abundant taxa between groups were assessed with ANCOM [36] and dsf-dr [37]. P values lower than 0.05 were considered statistically significant. 

Bacterial co-occurrence network and connection modules were computed with SparCC [38] using the *q2-SCNIC* plugin [39]. The co-occurrence network was analyzed and visualized with Cytoscape, version 3.9.1 [40].

## 3. Results

### 3.1. The Diversity of the Fecal Microbiota Changed through Time but Not according to Health Status

**Figure 1:**
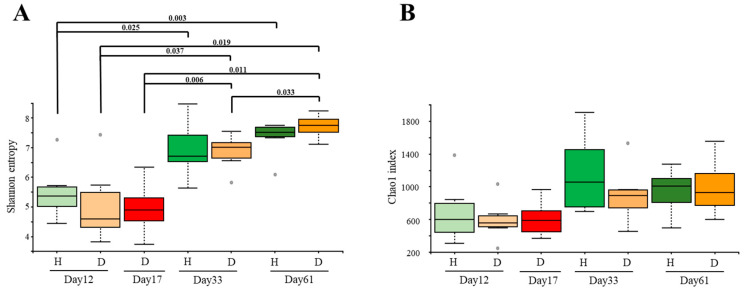
Alpha diversity of the fecal microbiota of calves that had a diarrhea episode (D, in orange-red colors) or not (H in different green colors) through time. (**A**) Shannon index and (**B**) Chao1 index were estimated at maximum depth of 58,940 for the groups under study. Intensity of colors indicates the sampling time (days 12, 33 and 61). Alpha diversity at the episode of diarrhea (D17) is red-colored. *p* values computed with pairwise non-parametric *t*-tests (999 random permutations) are depicted above each bar when it was lower than 0.05.

During the first month of life (from day 12 to day 33), there was an increase in alpha diversity in both H and D groups (*p* < 0.05) when measured by Shannon index (Figure 1A), while the mean microbial richness (Figure 1B) did not change, showing only a tendency to increase in the H group (Chao1 index, *p* = 0.06, Figure 1B). During the following month (day 33 to day 61), the microbial diversity remained stable in the H group but increased in the D group (*p* = 0.033; Figure 1A), whereas richness remained constant during this time in both groups. At the moment when diarrhea was observed in group D (D17), alpha diversity was as low as at D12 and lower than at D33 or D61 (*p* < 0.05, measured by Shannon index; Figure 1A). However, richness did not differ when comparing D17 with any other timepoint (Figure 1B, Chao1 index). On the other hand, when H and D groups were compared at the same sampling times, no differences in diversity were detected at any time point.

**Figure 2:**
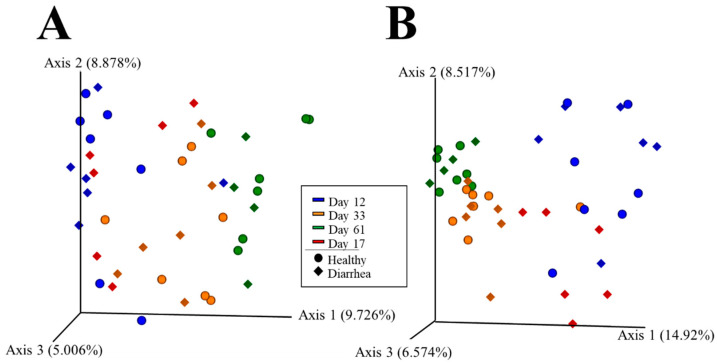
Beta diversity of the fecal microbiota of calves that had a diarrhea episode (D, in diamonds) or not (H, in spheres) through time. Principal Coordinate Analysis (PCoA) was computed with (**A**) Jaccard and (**B**) Bray Curtis dissimilarity indexes of the fecal microbiota of calves under study: day 12, in blue; day 33 in orange, day 61 in green; episode of diarrhea (D17) in red. Spheres and diamonds represent samples from H and D groups, respectively.

Beta diversity was assessed with Jaccard and Bray-Curtis dissimilarity indexes for H and D groups at the different sampling time points (Figure 2). In group H, the effect of time shaped the microbiota composition. The microbial communities at the three time points were different under both qualitative (Jaccard, Figure 2A) and quantitative (Bray–Curtis, Figure 2B) analyses, with an estimated percentage of explanation by the ‘time’ variable of 17.5% and 23.3% (Adonis R^2^, *p* = 0.001), respectively. Time also shaped the microbial communities in the D group in the qualitative analysis (Jaccard, Figure 2A), where only the bacterial communities found at the moment of diarrhea (D17) were still not different from those at D12, but differed from the microbiota diversity at day 33 or day 61. Nevertheless, the microbial diversity from the D group was different at every time point in the quantitative analysis (Bray–Curtis, Figure 2B). The percentage of explanation of these differences was 19% and 28.4% in Jaccard and Bray–Curtis analysis, respectively (Adonis function, *p* = 0.001). The pairwise group significances calculated with the PERMANOVA test with 999 random permutations are depicted in Supplementary Table S2. We performed two different tests, ANCOM and dsf-dr, in order to find the differentially abundant taxa changing through time where we only found a few taxa, probably due to the intra-individual variability within each group. The list of differentially abundant taxa found is provided in Supplementary Table S3.

**Figure 3:**
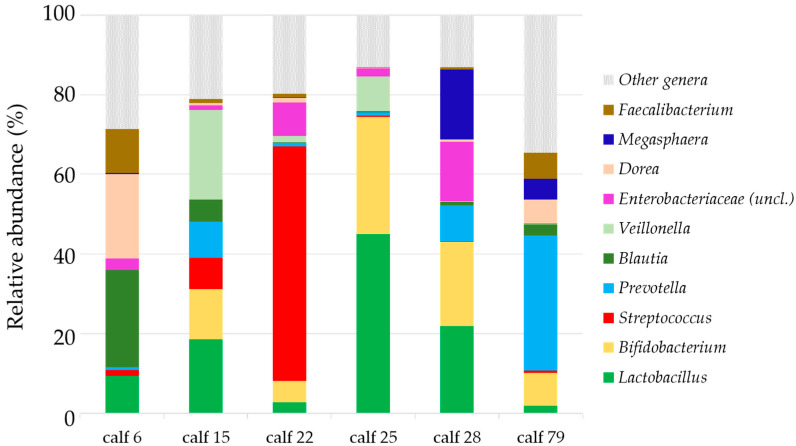
Percentage of relative abundance of genera from fecal microbiota of animals that had diarrhea. Taxa bar plot of the top 10 most relatively abundant genera found globally in the microbiota from animals that had diarrhea at day 17 (D17) is depicted. The genera are ordered by increasing global relative abundance from bottom to top.

When the microbiota diversity was compared between H and D groups at each of the sampled time points (Figure 2), no differences in beta diversity were found that could be associated with either a predisposition to disease (when comparing H12 vs. D12 group) or to potential changes that could have occurred after diarrhea and subsequent antibiotic treatment (considering H33 vs. D33 or H61 vs. D61). Accordingly, no differentially abundant taxa were found among groups, despite several tests being performed. Noteworthily, high variability within each group was found especially on days 12 and 17 as revealed in the dispersion found in the beta diversity analysis (Figure 2), which might have prevented the detection of significantly differential taxa between groups. Moreover, at the moment of pathology, we found that the microbiota showed a variable composition in each animal and was dominated by few specific taxa (D17 group most relatively abundant taxa, depicted in Figure 3), such as *Lactobacillus*, *Bifidobacterium*, *Streptococcus*, *Prevotella*, *Blautia*, *Veillonella*, *Enterobacteriaceae* (*uncl*.), *Dorea*, *Megasphaera* and *Faecalibacterium*.

### 3.2. The Core Microbial Composition Was Different Depending on the Health Status

**Figure 4:**
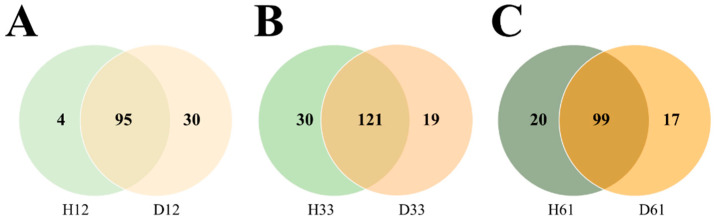
Venn’s diagram of the genera found in the core microbiota of calves that had a diarrhea episode (D, in orange) or not (H, in green). The number of core taxa found in the fecal microbiota of both groups was defined as taxa present in, at least, 80% of samples within a group at (**A**) day 12, (**B**) day 33 and (**C**) day 61. The full list of taxa shared and exclusively from each group at each timepoint is in Supplementary Table S4.

In order to overcome the problem of the high variability intra-group and reveal potential differences that might have been hidden, the core microbiota was studied. The core microbiota was calculated according to health status at each time point, with those genera present in at least 80% of samples. At the genus level, the core from each group was compared at each time point (Venn’s diagram, Figure 4). The full list of shared and health-status-specific genera is included in Supplementary Table S4. At all time points, most of the core taxa were shared between H and D groups (n = 95 at day 12, n = 121 at day 33 and n = 99 at day 61). Notably, the most relatively abundant genera (>1% relative abundance) composing the microbiota of all groups were shared among cores. The relative abundance of the most relatively abundant core genera for each group at all time points is shown in Supplementary Figure S1. On day 12, the core fecal microbiota showed to be dominated by the genus *Bacteroides*, accounting for 23% and 17% of the total relative abundance for D12 and H12 groups, followed by *Lactobacillus* and *Faecalibacterium* with 6% and 5% of relative abundance in both groups. The two most relatively abundant genera within the *Proteobacteria* phylum globally were *Escherichia* (2.1% in D12 and 0.3% in H12) and an unclassified genus from the *Enterobacteriaceae* family (5% in D12 and 2.1% in H12). At later ages, the most relatively abundant genera changed to be *Blautia* for both groups with similar abundances at day 33 (12%), although with a different tendency through time, since the mean relative abundance in H61 was 16% while in D61 was 10%. Other relatively abundant genera at these time points, were *Bacteroides* and *Prevotella* together with *Faecalibacterium* and an unclassified genus from *Ruminococcaceae*, although with similar abundances between H and D groups at each time point. 

Nevertheless, some differences between core genera from the H and D groups were observed (Figure 4). There were 30 genera exclusively found in the D12 core (not in H12), which could be related to predisposition to the disease. Of these 30 genera, 14 were not found in the H33 or H61 core, but three (*Lachnospira*, *Neisseria* and *Solibacillus*) were also part of the D33 and D61 core. Other genera specifically found in the D12 core (absent in the D33 and D61 cores) were *Peptostreptococcus*, *Erysipelothrix* and *Ochrobactrum*. On the other hand, four genera were found only in the H12 core, including *Epulopiscium*, which was absent later in life. 

### 3.3. Animals from Healthy or Disease Groups Showed Different Co-Occurrence Microbial Networks

In order to identify bacterial variations with biological relevance that could be related to the predisposition to diarrhea, a correlation network analysis at the genus level was performed on the microbiota from H and D groups at day 12. We considered only nodes (genera) with a degree > 1 (more than one edge) and highly correlated (r ≥ 0.85) for the analysis. The topology of the network was different when it was built with samples from the D group (D-network) or from the H group (H-network). The D-network was more complex, as it was composed of 62 nodes and 111 edges with a diameter of 10, while the H-network had 49 nodes connected by 79 edges and a diameter of 8. The nodes from the D-network showed a greater level of connection with a maximum degree of 13, in contrast to the H-network which was 7. 

**Figure 5:**
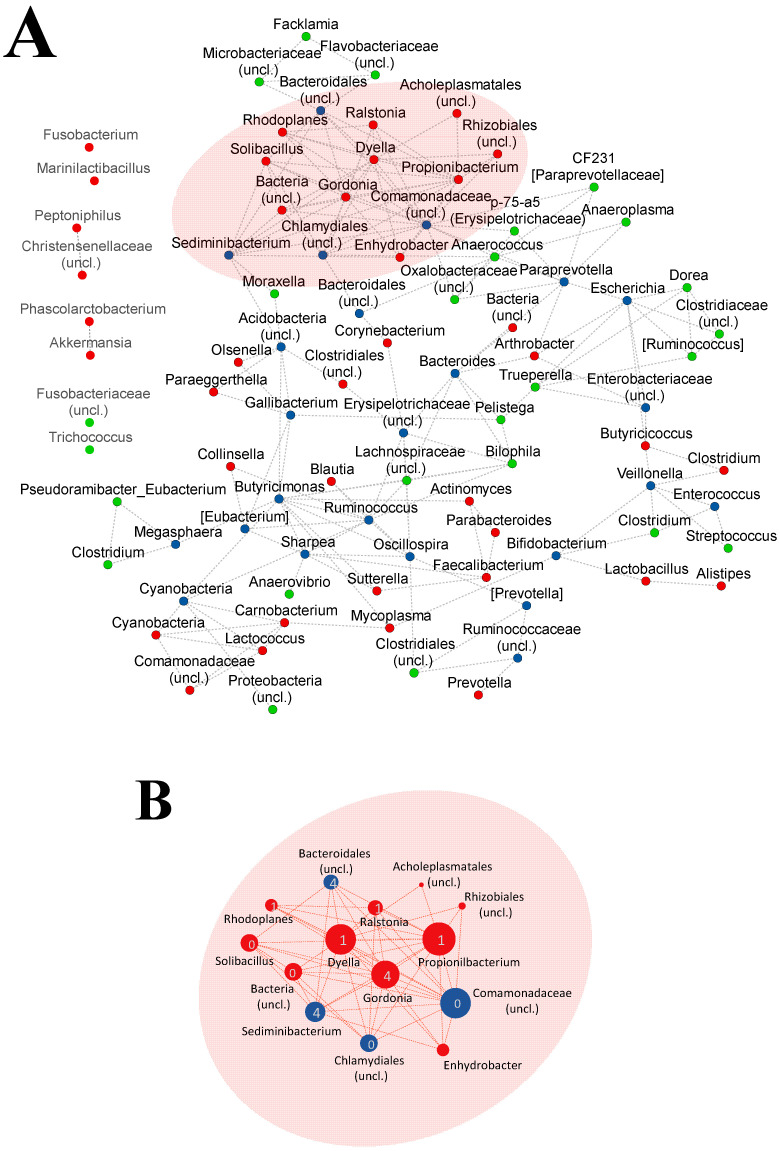
Correlation network analysis of microbial components from the fecal microbiota of calves that had a diarrhea episode (D) or not (H) at day 12. (**A**) Combined co-occurrence network of the genera found in the fecal microbiota of calves from D and H groups at day 12. Only associations with an r value (SparCC correlation value) greater than 0.85 and nodes with a degree higher than 2 are represented. (**B**) The most connected component of D-network is represented separately for better visualization. The modules are indicated with numbers inside the nodes and the size of the nodes is proportional to their degree (number of connections), which ranges from 2 to 13. Nodes are colored according to the networks they belong to, with green nodes for H network and red nodes for D network; blue nodes correspond to genera found in both networks.

The two networks were overlapped to highlight differential nodes and components (Figure 5A). We found 24 nodes shared between the two networks, mainly including the highest relatively abundant taxa identified at this time point (*Bacteroides*, *Bifidobacterium*, [*Prevotella*], *Enterobacteriaceae* (*uncl*.), *Ruminococcaceae* (*uncl*.), *Escherichia* and *Enterococcus*). Moreover, 25 and 38 nodes were detected specifically in the H- and D-network, respectively. The most correlated nodes were different in each network, highlighting the differences between microbial compositions (Supplementary Table S5). *Propionobacterium*, *Comamonadaceae* (*uncl*.), *Dyella* and *Gordonia* (with a degree ≥ 11) from the D-network belonged to three different modules (areas of the network strongly correlated). These three modules (modules D-0, D-1 and D-4) were grouped into a single connected component in the network, containing most of the highly-connected nodes as shown in Figure 5B. Importantly, most of these genera from this connected component from the D-network were absent or with a low degree of correlations in the H network. Interestingly, *Solibacillus* and *Enhydrobacter*, both found within this D-network connected component, were found among the D-group-specific core microbiota at day 12. We did not find a single highly-connected component in the H network, as the most-connected genera were grouped into six different modules. In fact, the top three most-correlated genera (*Anaerococcus*, *Oscillospira* and *Ruminococcus*) belonged to two separate network components. Interestingly, most of these highly-connected genera (degree ≥ 5) from the H network were absent or had a lower degree (degree ≤ 3) in the D-network, with the exception of *Comamonadaceae* (*uncl*.) and *Butyricimonas*, which were found also in the D-network with a higher degree (12 and 7, respectively). 

## 4. Discussion

Bacterial communities represent a major portion of the fecal material, and their presence is crucial for the health of mammals, including dairy cows [41]. In this study, we analyzed the fecal microbiota from calves entering a commercial operation until weaning, in order to assess the connection between early-life diarrhea episodes in the farm and the fecal microbiota composition. Although individual variability was high, several changes in the fecal microbiota were found before the episode of diarrhea, which can be related to predisposition. In addition, different diversity dynamics were observed after diarrhea, which can be related to the disease and/or the antibiotic treatment, a common procedure in most farms [2,4].

The alpha diversity of the fecal microbiota of calves that remained healthy throughout the study (H group) increased during the first month of life when it seemed to stabilize. This was observed before in pre-weaned calves where the microbial richness stabilized 1 week before weaning [42]. Nevertheless, we detected differences in beta diversity between H33 and H61 groups, demonstrating that the microbial populations are still fluctuating during the second month of life. In the case of animals that had diarrhea (D group), we did not find the same tendencies, because the alpha-diversity increased continuously until the end of the study, suggesting that stability might not be reached in these animals due to the diarrhea episode and/or the subsequent antibiotic treatment. It is known that higher stability confers higher resilience to external perturbations such as those produced by pathogens [43]. The alpha diversity at the moment of diarrhea (D17) was not different from D12 or H12, but it was lower than D33 or H33, which might be related to the episode of diarrhea but also to the instability of the immature microbiota at these ages [44]. The low alpha diversity seen at this time point could also be caused by the overgrowth of specific bacterial species that can occur during the so-called “window of susceptibility”, when the protective antibody titers are low, as previously observed in calves’ upper respiratory tract [45]. Since no qualitative differences were found between the bacterial populations in D17 or D12 in the beta diversity analysis, it seems that the microbiota in D17 is still immature, colonized mainly with the communities present at early-life (D12). Unfortunately, we could not get the samples from healthy animals at this timepoint (day 17), limiting our conclusions. Nonetheless, these different tendencies were also reflected in the correlation network analysis when H and D networks were compared, where we found different associations as early as the day of arrival (day 12 of life). Interestingly, the most correlated genera varied between groups, showing different relationships in the bacterial populations of the microbiota regarding the health status of the animals. Altogether, these results suggest that the stabilization of the microbiota in early life (achieved in H33 but not in D33), may promote better health [6,46]. 

On the other hand, we were not able to detect changes in alpha and/or beta diversity when the health status of the animals was evaluated at each time point. The intra-variability observed in the groups made this analysis difficult. The composition of the microbiota can be influenced by many factors that are inherent to animal production, such as the environment, the genetics of the animals or the diet [8], and can be confounders in the analyses. The variability in this study is probably caused by the different origins of the animals, since the management on the site of the study, including the diet, was common to all the animals. In fact, this high dissimilarity within fecal microbiota from each group has been also documented before for calves. For example, Mayer et al. demonstrated by single-strand conformation polymorphism, that similarity in the microbiota of calves from the same group decreases until 14 days of life and represented an average of only 14% of similarity at 42 days of life [47]. This observed variability together with the limited number of samples available in this study, complicated finding particular taxa associated with the episode of diarrhea. However, for D17 samples, we found some taxa in each animal that might be related to diarrhea, such as *Veillonella*, an oral pathogen in humans [48] hardly assessed in calves, identified only in the small intestinal microbiome [44]; or *Enterobacteriaceae*, as members of this family were found associated with diarrhea in different animal species, including calves [49]. We also identified an over-increased relative abundance of *Streptococcus* in some samples from the D17 group, in agreement with Henessy et al., where *Streptococcus* was found in significantly higher abundance in diarrheic calves [12]. Importantly, Ma et al. also defined the presence of *Streptococcus* and *Dorea* as a possible marker for diarrheic gut microbiota [6], which were among the taxa we detected at the moment of pathology (D17). The main technical difference between this present and previous studies is the region from the 16S rRNA gene used to perform the bacterial characterization, which has been described as of particular importance when comparing the conclusions raised between studies [50]. The fact that some of the main taxa found in this study were associated with disease coincides with these previous analyses [6,12], validates and reinforces the conclusions. On the other hand, among relatively abundant genera found in D17, we found genera frequently found in the commensal gastrointestinal bacteria [13,44,51,52], and we cannot discard the presence of pathogenic strains within these genera which may be the cause of the observed disease. In addition, other etiological agents, such as viruses, can cause diarrhea in calves [1,3] and were not assessed in this study. Nevertheless, the unbalanced microbiota observed at the moment of diarrhea is in agreement with other studies that correlate microbial dysbiosis with disease in calves [49] and other mammals [53]. 

To aid in the detection of possible markers for diarrhea predisposition avoiding the intra-group variability, the core microbiota of the groups was analyzed allowing to qualitatively compare the microbial composition between groups. Interestingly, among the 30 genera found exclusively in D12, *Solibacillus*, *Lachnospira* and *Neisseria* were detected specifically in the D core at every timepoint. Interestingly, *Solibacillus* was also found as a highly correlated connected component in the D-network, highlighting its potential role; unfortunately, there are scarce reports on this genus, which prevents us from further hypothesizing on its particular role in disease. There were other taxa detected only in D12 and not in D33 or D61. The absence of these taxa at later time points may be associated with the episode of diarrhea, but also with the antibiotic treatment. Only three out of these taxa were classified to genus level: *Peptostreptococcus*, previously described as diarrhea-related [12]; *Erysipelothrix*, a known pathogen of humans and other mammals [54]; and *Ochrobactrum*, not previously described in calves’ gastrointestinal tract. Although these genera were found among the low abundant microbiota taxa (<1% relative abundance), they might play important roles in the whole microbial community that need to be elucidated. In the case of animals undergoing diarrhea, a cluster of highly connected genera was identified in D-network, that were not present in H-network. Despite most of these genera also being members of the shared core microbiota, we cannot discard their role in the subsequent development of diarrhea, where they might interact differently in the microbial network. The main objective for defining a common core among animals from the same health status, was to identify the microorganisms of particular importance for host function that might be altered differentially. We also built correlation networks for both groups before diarrhea development, to find the bacterial members predisposing these calves to disease. However, we did not find the same genera when we compared the members of the exclusively disease-core at early life (D12) with the major components (highly-connected nodes) of the D-network, which may seem contradictory results. Noteworthy, these strategies use different input data (qualitative for core analysis and quantitative for SparCC) and thus, the results can be complementary. On one hand, the core analysis revealed an exclusive genus present at high frequency (>80% of the animals), which might be related to essential host functions despite being in low abundance. The assumption that high abundant species are more relevant for the whole community has been challenged over the years with evidence that rare taxa can be just as important as widespread taxa [55,56,57]. On the other hand, we used SparCC to robustly characterize the two microbial environments (health vs. disease) avoiding spurious correlations [39,58]. The network-based approach unveiled different components of each network that demonstrate distinct microbial interaction patterns associated with the future development of diarrhea. 

## 5. Conclusions

In this study, we found different dynamics in the alpha diversity of the calves’ fecal microbiota through life in healthy and diarrheic calves. In the healthy group, the microbial populations seemed to stabilize earlier when compared to the tendencies observed in the disease group, with disadvantageous alterations present in the bacterial network linked to disease. Although we did not find differentially abundant taxa when comparing these two groups at each time point, we suggest that differences in the correlations between microbial populations (especially the low abundant species) at early life may predispose to diarrhea. Further analysis should include a higher number of animals to avoid the high degree of individual variability of the early fecal microbiome observed and confirm these results. Still, there is room for further assessment to identify the microbial populations that must be present in the fecal microbiota to benefit long-term health.

## Figures and Tables

**Table 1:** Calves’ farm of origin and age at sampling of the calves enrolled in the study.

Calf ID	Farm of Origin	Group	Age at Sampling (Days)			
			Reception	Disease	First Month	Second Month
1	1	Healthy	15	Not sampled	35	63
4	2	Healthy	7	Not sampled	34	62
5	3	Healthy	8	Not sampled	35	63
6	4	Diarrhea	11	18	31	59
7	5	Healthy	14	Not sampled	34	62
8	6	Healthy	13	Not sampled	33	61
9	6	Healthy	12	Not sampled	32	60
15	7	Diarrhea	11	16	31	59
22	8	Diarrhea	12	16	32	60
25	5	Diarrhea	13	17	33	61
26	9	Healthy	11	Not sampled	31	59
28	5	Diarrhea	10	16	30	62 *
79	6	Diarrhea	13	17	33	60 *

* Calves 28 and 79 sequences at second month were not included in the study due to low number of reads.

## Data Availability

The entire sequence dataset is available in the NCBI database, BioProject ID PRJNA799186 available at http://www.ncbi.nlm.nih.gov/bioproject/799186, uploaded on 21 January 2022.

## Supplementary Materials

The following supporting information can be downloaded at: https://www.mdpi.com/article/10.3390/vetsci9090463/s1, Figure S1: Taxa bar plot of the relative abundance of genera over 1% in relative abundance found in the core microbiota at each time-point of healthy (H) and diarrhea (D) groups: (**A**) day 12, (**B**) day 33 and (**C**) day 61. Table S1: Number of reads per sample. Table S2: Beta diversity group significance of the microbiota found in H and D groups at different time points, computed through PERMANOVA test using 999 random permutations. Table S3: Mean group relative abundance of the differently abundant taxa found with ANCOM and dsf-dr between time points. Table S4: Genera found within the core-microbiota (computed at 80%) of the calves under study at days 12, 33 and 61. Genera found in H and D groups are depicted as shared, while genera found only in one group are depicted as exclusive. Table S5: Correlation of genera found in H and/or D networks at day 12 with a value of correlation r ≥ 0.85, with degree (number of connections) and the module they belong (group of nodes) depicted.
