# Supplementary material for: Longitudinal Study of Fecal Microbiota in Calves with or without Diarrhea Episodes before Weaning
Source: Vet Sci. 2022 Aug 29;9(9):463. doi: 10.3390/vetsci9090463 (PMC9503950; doi:10.3390/vetsci9090463)
Supplement: Supplementary file 1 [file vetsci-09-00463-s001.zip › Supplementary_Figure_S1B.pdf]

B

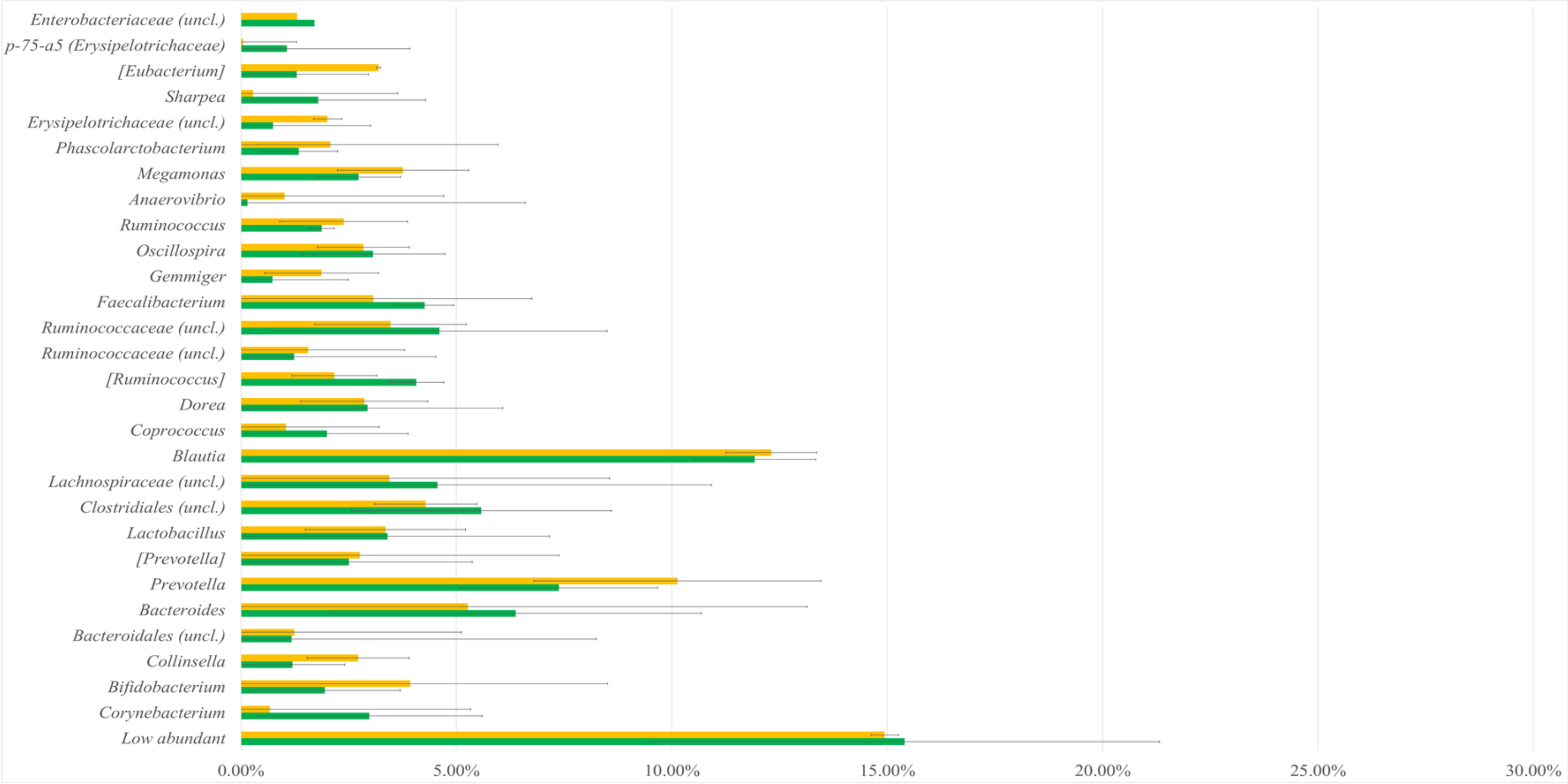

Supplementary Figure S1. **Taxa bar plot of the relative abundance of genera over 1% in relative abundance found in the core microbiota at each time-point of calves suffering a diarrhea episode (D, in orange) or not (H in green).** The depicted genera were found at (A) day 12, (B) day 33 and (C) day 61.
