# Supplementary figures and images for: Longitudinal Study of Fecal Microbiota in Calves with or without Diarrhea Episodes before Weaning
Source: Vet Sci. 2022 Aug 29;9(9):463. doi: 10.3390/vetsci9090463 (PMC9503950; doi:10.3390/vetsci9090463)

A

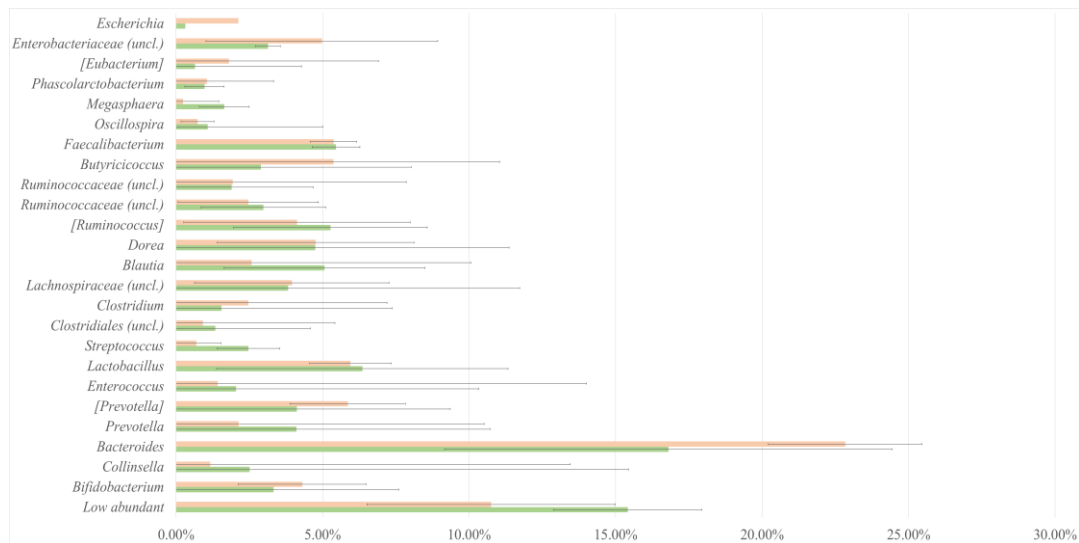

B

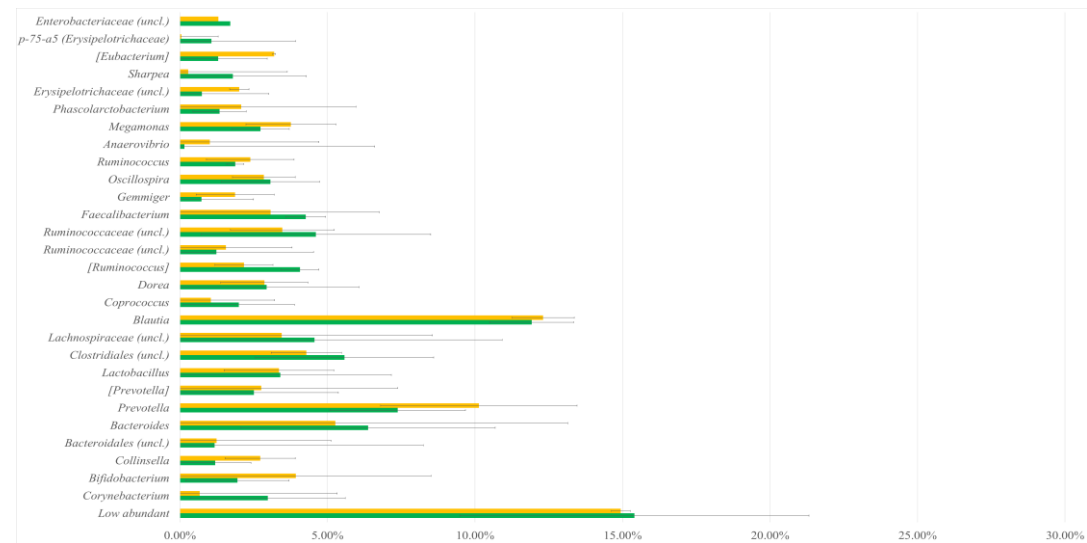

C

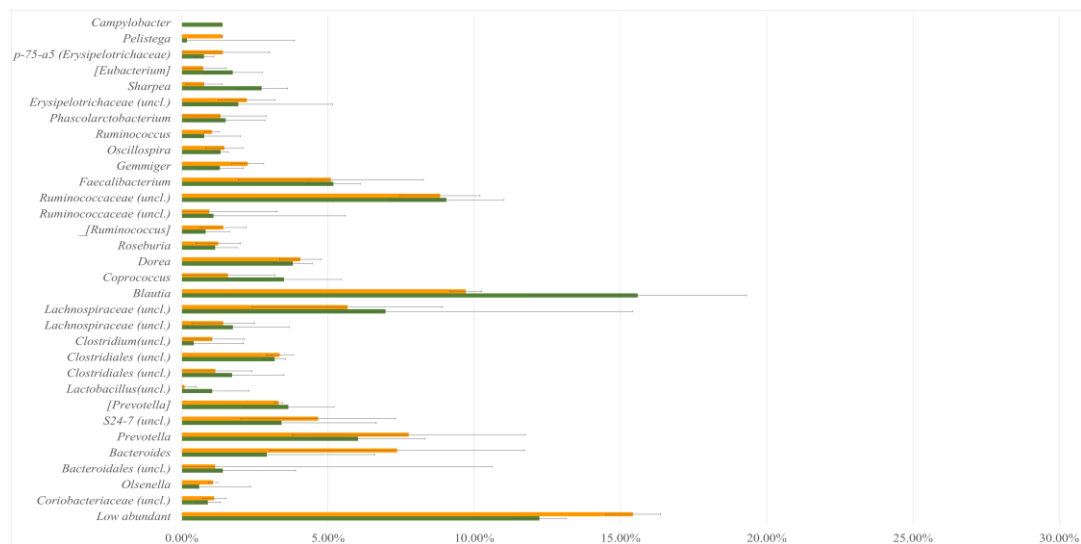

Supplement: Supplementary file 1 [file vetsci-09-00463-s001.zip › Supplementary_Figure S1.pdf]
